# Supplementary material for: Unintentional Tobacco Smoke Exposure in Children
Source: Int J Environ Res Public Health. 2022 Jun 9;19(12):7076. doi: 10.3390/ijerph19127076 (PMC9222355; doi:10.3390/ijerph19127076)
Supplement: Supplementary file 1 [file ijerph-19-07076-s001.zip › ijerph-1769572-supplementary.pdf]

Unintentional Tobacco Smoke Exposure in Children Supplementary Materials

Table S1. Participants in smoking homes by temperature quartile for all participants that completed a questionnaire.

Temp Quartiles	-15°C to -5°C	-4°C to 5°C	6°C to 16°C	17°C to 27°C
Number of participants[a]	49	38	19	112
Number of participants in smoking homes (%)	14 (28.6)	5 (13.2)	3 (15.8)	21 (18.8)
Average smokers per smokers' home	2.1	3.4	2.7	1.3

[a]15 participants missing due to incomplete temperature data

Table S2. Participants in smoking homes by temperature quartile for participant dyads that completed a questionnaire and provided a urine sample.

Temperature Range	-15°C to -5°C	-4°C to 5°C	6°C to 16°C	17°C to 27°C
Number of participants[b]	31	25	12	60
Number of participants in smoking homes (%)	9 (29.0)	3 (12.0)	4 (33.3)	16 (26.7)
Average smokers per smokers' home	2.7	2.3	2.8	1.1
Participants above 30 ng/mg CCR cutoff (%)	4 (12.9)	4 (16.0)	2 (16.7)	8 (13.3)

CCR median (IQR)	0.00 (0.00 to 12.24)	2.17 (0.00 to 10.48)	3.88 (1.09 to 6.59)	1.61 (0.00 to 7.21)

CCR = cotinine-creatinine ratio

[b]15 participants missing due to incomplete temperature data
